# Hypoxia-Induced Kidney Injury in Newborn Rats

**DOI:** 10.3390/toxics11030260

**Published:** 2023-03-11

**Authors:** Yi-Ting Chu, Bo-Hau Chen, Hsin-Hung Chen, Jui-Chen Lee, Tzu-Jiun Kuo, Hsiang-Chin Chiu, Wen-Hsien Lu

**Affiliations:** 1 Department of Pediatrics, Kaohsiung Veterans General Hospital, Kaohsiung 813414, Taiwan; 2 Department of Pediatrics, Taoyuan Armed Forces General Hospital, Taoyuan 32551, Taiwan; 3 Department of Medical Education and Research, Kaohsiung Veterans General Hospital, Kaohsiung 813414, Taiwan; 4 Department of Pediatrics, Pingtung Veterans General Hospital, Pingtung 91245, Taiwan; 5 School of Medicine, National Yang-Ming University, Taipei 11221, Taiwan; 6 Institute of Biomedical Sciences, National Sun Yat-sen University, Kaohsiung 804201, Taiwan

**Keywords:** apoptosis, fibrosis, hypoxia, kidney injury, neonate, oxidative stress

## Abstract

Exposure to hypoxia during the early postnatal period can have adverse effects on vital organs. Neonatal Sprague–Dawley rats housed in a hypoxic chamber were compared to those in a normoxic chamber from postnatal days 0 to 7. Arterial blood was collected to evaluate renal function and hypoxia. Kidney morphology and fibrosis were evaluated using staining methods and immunoblotting. In the kidneys of the hypoxic group, protein expressions of hypoxia-inducible factor-1 were higher than those in the normoxic group. Hypoxic rats had higher levels of hematocrit, serum creatinine, and lactate than normoxic rats. Body weight was reduced, and protein loss of kidney tissue was observed in hypoxic rats compared to normoxic rats. Histologically, hypoxic rats showed glomerular atrophy and tubular injury. Renal fibrosis with collagen fiber deposition was observed in the hypoxic group. The expression of nicotinamide adenine dinucleotide phosphate oxidases was enhanced in the kidneys of hypoxic rats. Proteins involved in apoptosis were upregulated in the kidneys of hypoxic rats. An increase in the expression of pro-inflammatory cytokines was also observed in the kidneys of hypoxic rats. Hypoxic kidney injury in neonatal rats was associated with oxidative stress, inflammation, apoptosis, and fibrosis.

## 1. Introduction

Hypoxia in early postnatal life leads to adverse effects on growth and development. The duration of hypoxia and age at the time of exposure are also associated with postnatal organ growth and development [1,2]. This has been supported by many clinical and experimental studies [3]. Neonatal hypoxia can occur in various conditions, such as congenital heart disease, bronchopulmonary dysplasia, pulmonary hypertension, airway obstruction, and sepsis. The effects of neonatal hypoxia on body weight and the brain, heart, and lungs have been investigated [4,5,6,7,8]. The kidney is also an important organ that requires high perfusion and oxygen supply, and it is sensitive to hypoxic injury [9,10]. However, few studies have focused on neonatal hypoxia, which is a common clinical condition in newborns with affected kidney morphology, kidney injury, and long-term effects.

Nephrogenesis in humans starts at 9–10 weeks of gestation, continues rapidly between 18 and 32 weeks, and is completed between 32 and 35 weeks [11]. Most preterm neonates are born during active nephrogenesis, resulting in a reduced number of nephrons. A lower nephron number at birth may not lead to kidney dysfunction, but the kidney is vulnerable to the ex-utero environment and nephrotoxic insults, such as acute or chronic kidney injury, which increases the risk of progressive kidney disease in later life. Preterm infants can be exposed to hypoxia during the early postnatal period owing to various respiratory and cardiorespiratory insufficiencies. Low oxygen supply and high oxygen demand make the kidney vulnerable to hypoxia; thus, it might have adverse effects on renal development and cause poor kidney-related outcomes. Compared with kidney development in humans, the process of nephrogenesis in rats is completed within 10 days after birth [12,13,14]. Thus, day 7 of postnatal development in the rat kidney can be regarded as a model for preterm infants born at 34–36 weeks of gestation because the renal tissue resembles the human preterm kidney [15].

In this study, we observed a rodent model from birth to postnatal day 7, which represents the neonatal period [16]. Hypoxic exposure immediately after birth raises the level of stress imposed on the immature kidneys and mimics the common condition of preterm newborns. Acute kidney injury (AKI) usually occurs in neonatal intensive care units and is a crucial risk factor for the development and progression of chronic kidney disease [17]. During the neonatal period, there is no consensus on the definition of AKI based on serum creatinine, and creatinine values are unreliable in the first postnatal week, especially in preterm infants [18]. This means that we might underestimate kidney damage after neonatal hypoxia. Although hypoxia is one of the most common causes of acute and chronic kidney injury, the precise mechanisms of hypoxia-induced kidney injury remain poorly understood [19]. Zangaladze et al.’s study mimicked neonatal intermittent hypoxia after birth and found kidney damage with associated elevations of vasoconstrictors [20], and Plotnikov et al. explored acute kidney injury with severe hypoxia (8% O_2_) for 2 h in the neonatal rat [15]. Differently from those two studies, our study focused on persistent, not intermittent, neonatal hypoxia mimicking clinical condition, such as congenital heart disease or bronchopulmonary dysplasia, and further investigated the effects and molecular mechanism of kidney injury.

## 2. Materials and Methods

### 2.1. Experimental Design

All animal research protocols were approved by the Institutional Animal Care and Use Committee of Kaohsiung Veterans General Hospital (identification code: 2018-A006, 2022-A032, and IACUC-2301-2312-22100; date of approval: 12 May 2017, 21 May 2021, and 30 November 2022). Pregnant Sprague–Dawley rats were purchased from BioLASCO Taiwan Co., Ltd. (Taipei, Taiwan) and acclimatized to the animal facility for a week. These rats were maintained under a temperature of 23 to 24 °C and a 12 h light and 12 h dark cycle with food and water. The rat pups, within 24 h of birth, and their mother were transferred into the C-Shuttle Glove Box, which was used to provide a normobaric hypoxic environment (13% oxygen), while the control group remained in room air.

The rat pups were divided into the following two groups: (1) normoxic group (room air, *n* = 8), (2) hypoxic group (13% oxygen, *n* = 6). The experiment was performed on both-gender 7-day-old rat pups, weighing 11–16 g. Rat pup weights were recorded on postnatal days 0 and 7, and then, they were euthanized via decapitation after isoflurane-induced (Panion & BF Biotech Inc., Taipei, Taiwan) narcosis.

### 2.2. Arterial Blood Gas Measurements

Blood was rapidly collected using a Critical Care Blood Collection System tube (Becton Dickinson, Franklin Lakes, NJ, USA) and analyzed within 30 min using the Epoc^®^ blood analysis system (Siemens Healthineers, Erlangen, Germany). We measured the levels of pH, glucose, creatinine, lactate, partial pressure of carbon dioxide, partial pressure of oxygen (pO_2_), total carbon dioxide concentration of oxygen saturation, sodium, potassium, chloride, calcium, hematocrit, hemoglobin, bicarbonate, base excess of the extracellular fluid, blood base excess, potassium, and anion gap.

### 2.3. Tissue Preparation for Histological Studies

Kidney tissues were fixed with 10% neutral buffered formalin and embedded in paraffin (Taiwan Burnett International Co., Ltd., Taipei, Taiwan). The sections were cut to 4 μm thickness. Before staining, the paraffin-embedded kidney sections were deparaffinized and hydrated.

### 2.4. Morphology of the Kidney and Quantitative Assessment of Tubular Injury

Kidney sections were stained with hematoxylin-eosin (HE) (Leica Biosystems, Wetzlar, Germany) and periodic acid-Schiff (PAS) stains (395B, Sigma-Aldrich, St. Louis, MA, USA) to evaluate the kidney morphology. Tubular injury was scored semi-quantitatively by examining at least 15 fields of PAS-stained sections. Tubular injury was defined as loss of the brush border, tubular atrophy, tubular cast formation, tubular dilation, thickening of the tubular basement membrane, and sloughing of the tubular epithelial cells. The evaluation was scored according to the following scoring system: score 0, no tubular injury; score 1, <10% of tubules are injured; score 2, 10–25% of tubules are injured; score 3, 25–50% of tubules are injured; score 4, 50–74% of tubules are injured; and score 5, >75% of tubules are injured [21].

### 2.5. Collagen Fibers and Collagen Volume Fraction (CVF) Detection

We used picrosirius red staining (395B, Sigma-Aldrich) and Masson trichrome staining (HT-15, Sigma-Aldrich) to detect collagen fibers in the kidney tissue. Before staining, sections were incubated for 1 h at 56 °C in Bouin’s reagent (HT10132, Sigma-Aldrich). Following that, the sections were incubated in picrosirius red solution for 2 h before being washed in 1% acetic acid and mounted after dehydration. We randomly selected 10 fields for each sample and calculated them using ImageJ software, version 1.46 (Media Cybernetics, Inc., Rockville, MD, USA). The Masson trichrome staining of kidney sections was performed using a ready-to-use kit. The analysis was performed by calculating the CVF, which is the percentage of the collagen-positive blue area, in relation to the total tissue area.

### 2.6. Immunohistochemical Analysis

Kidney sections were placed in a 0.01 M sodium citrate buffer at 95–100 °C for 20 min and then cooled to room temperature. The sections were incubated in a peroxidase blocking solution (Novolink Polymer detection system, Leica Biosystems, Wetzlar, Germany) for 30 min, in a protein block for 30 min, and incubated with anti-neutrophil gelatinase-associated lipocalin (NGAL) (#ab63929, Abcam, Cambridge, UK) and anti-Klotho (28100-1-AP, Proteintech Group, Inc., Rosemont, IL, USA) antibodies at 4 °C overnight. The next day, the sections were incubated with Novolink polymer for 10 min, reacted with 3,3′-diaminobenzidine, counterstained with hematoxylin, and mounted after dehydration. The slides were photographed using an Olympus microscope (BX51, Olympus, Tokyo, Japan) equipped with a charge-coupled device imaging system (DP74, Olympus). Quantitative analysis was performed using the count and measure tool of ImageJ. The average optical density (AOD = integrated optical density/area) was used in statistical analysis.

### 2.7. Protein Extraction and Determination

Kidney tissues were lysed with a protein extraction reagent containing a lysis buffer (C2978, Sigma-Aldrich), a protease inhibitor cocktail (TAAR-BBI2, Tools Biotech, Taipei, Taiwan), a phosphatase inhibitor cocktail 2 (P5726, Sigma-Aldrich), and a phosphatase inhibitor cocktail 3 (P0044, Sigma-Aldrich). Lysates were sonicated with TissueLyser II (QIAGEN, Venlo, Netherlands). The lysates were incubated on ice for an hour and centrifuged at 13,000 rpm for 30 min at 4 °C; the supernatant was collected. Proteins were quantified using the Bradford protein assay (Coomassie Plus protein assay reagent, Thermo Fisher Scientific, Waltham, MA, USA).

### 2.8. Western Blot Analysis

Protein extracts were subjected to a TGX FastCast Acrylamide kit (Bio-Rad Laboratories, Inc. Hercules, CA, USA) and transferred to a polyvinylidene difluoride membrane (NEF1002001PK, PerkinElmer, Waltham, MA, USA). The membrane was blocked in PBST buffer with 5% nonfat milk and 5% bovine serum albumin before being incubated with anti-hypoxia-inducible factors, alpha subunit (HIF-1α) (20960–1AP, Proteintech), anti-heme oxygenase-1 (HO-1) (ADI SPA-895, Enzo Biochem, Inc., Farmingdale, NY, USA), anti-NGAL (ab63929, Abcam), anti- nicotinamide adenine dinucleotide 3-phosphate (NADPH) oxidase subunit p22-phox (P22) (sc271968, Santa Cruz Biotechnology, Dallas, TX, USA), NADPH oxidase subunit p47-phox (P47) (sc-17845, Santa Cruz), anti- NADPH oxidase 2 phox (NOX2) (ab129068, Abcam), anti- NADPH oxidase 4 phox (NOX4) (14347–1-Ig, Proteintech), anti-collagen type I (67288–1-Ig, Proteintech), anti-fibroblast growth factor 23 (FGF23) (Ls-C411984, Lifespan Biosciences, Seattle, WA, USA), phospho-p38 mitogen-activated protein kinase (MAPK) (Thr180/Tyr182) (p-P38) (#9211, Cell signaling, Danvers, MA, USA), p38 MAPK (P38) (9212S, Cell Signaling), anti-caspase-9 (GTX112888, GeneTex, Irvine, CA, USA), anti-cleaved caspase-3 (#9664, Cell Signaling), anti-tumor necrosis factor-α (TNF-α) (PA1079, Boster biological technology, Pleasanton, CA, USA), anti-nuclear factor kappa B (NF-κB) (10745–1-AP, Proteintech), anti-actin (MAB1501, Merck KGaA, Darmstadt, Germany), anti-alpha tubulin (ab7291 Abcam), and anti-GAPDH (60004–1-Ig, Proteintech) antibodies. Peroxidase-conjugated anti-mouse or anti-rabbit antibodies were used as the secondary antibodies. Signals were visualized using the SuperSignal™ Western Blot Substrate Bundle (A45917, Thermo Fisher Scientific), and images were obtained using the ChemiDocTM MP Imaging System (Bio-Rad). Images were analyzed using Image Lab, version 6.0 (Bio-Rad).

### 2.9. Statistical Analysis

Values are presented as mean ± standard deviation. A Mann–Whitney U test was used to analyze the data, and statistical significance was set at *p* < 0.05. Statistical analyses were performed using IBM SPSS Statistics, version 20 (IBM, Armonk, NY, USA), and GraphPad Prism, version 6.01 (GraphPad Software. Inc., San Diego, CA, USA, www.graphpad.com (20 February 2022)).

## 3. Results

### 3.1. Induction of Hypoxia in Neonatal Rats and Hypoxia Animal Model Establishment

**Figure 1:**
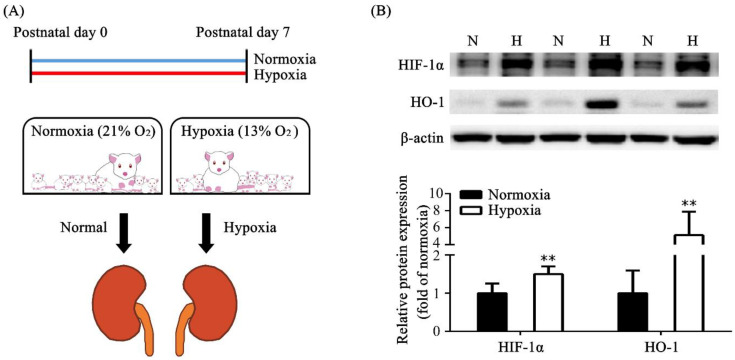
Neonatal rat model of the effect of hypoxia on the kidney after 7 postnatal days. (**A**) Our newborn rat model was used to study the effect of hypoxia on the kidney. (**B**) Protein expressions of HIF-1α and HO-1 in the kidney tissue of the normoxic and hypoxic groups are shown. The values are presented as mean ± standard deviation, ** *p* < 0.01, versus the normoxic group. The *p*-values were estimated via Mann–Whitney U test (*n* = 6). N, normoxia; H, hypoxia; HIF-1α, hypoxia-inducible factors, alpha subunit; HO-1, heme oxygenase-1.

In our experiment, rat pups were divided into a “normoxic group” exposed to ambient air (21% oxygen) with their mother after birth and a “hypoxic group” exposed to a normobaric hypoxic environment (13% oxygen) with their mother after birth (Figure 1A). The normoxic group had eight rat pups, and the hypoxic group had six rat pups. We continued the hypoxic environment for 7 days and then explored the effect on kidney tissue. According to Western blot analyses, we found that HIF-1α expression was significantly increased in the hypoxic group compared with that in the normoxic group. The protein level of HO-1 was also significantly increased in the hypoxic group compared with that in the normoxic group (Figure 1B).

### 3.2. Blood Gas Analysis

Blood gas analysis was performed to validate the hypoxia model. The arterial blood gas values at 7 days after exposure to different concentrations of oxygen are shown in Table 1. pO_2_ values were 27.77 ± 6.72 mmHg in the hypoxic group and 56.68 ± 4.09 mmHg in the normoxic group. The oxygen saturation was significantly lower in the hypoxic group than in the normoxic group. In hypoxic rats, significant polycythemia developed, and hematocrit levels were significantly higher than those in normoxic rats. Elevated creatinine and lactate levels were observed in the hypoxic group compared with the normoxic group. There was a significant elevation in potassium and chloride levels but no change in sodium and calcium levels in the hypoxic group. There was no difference in the pH value, pCO_2_, bicarbonate, and anion gap values between the groups.

### 3.3. Effect of Neonatal Hypoxia on Kidney Tissue

**Figure 2:**
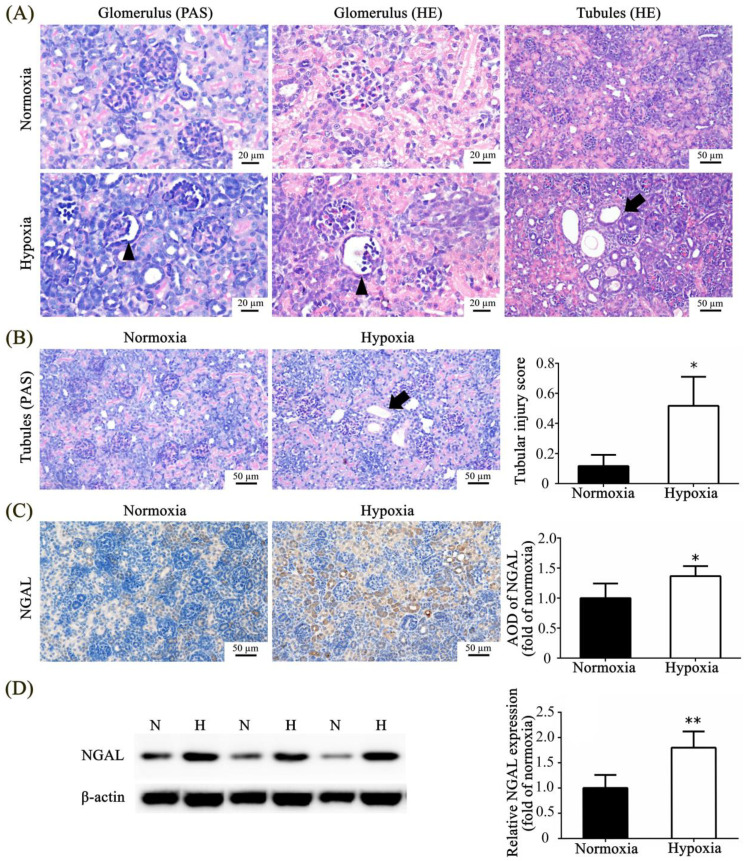
Neonatal hypoxia-induced glomerular and tubular injuries on postnatal day 7. (**A**) Representative PAS- and HE-stained images of the glomerulus (arrow head) under 400× magnification (scale bar, 20 μm) and representative HE-stained images of tubules (arrow) under 200× magnification (scale bar, 50 μm) in the kidneys. (**B**) PAS staining images show the tubules (arrow) under 200× magnification (scale bar, 50 μm) and the tubular injury score. Immunohistochemical staining (**C**), immunoblotting (**D**), and quantitative analysis of NGAL. The values are presented as mean ± standard deviation, * *p* < 0.05, ** *p* < 0.01 versus the normoxic group. The *p*-values were estimated via Mann–Whitney U test (*n* = 6). N, normoxia; H, hypoxia; PAS, periodic acid Schiff; HE, hematoxylin and eosin; NGAL, neutrophil gelatinase-associated lipocalin; AOD, average optical density.

We observed the morphology of glomeruli and tubules in the normoxic and hypoxic groups using PAS and HE staining methods. The glomerulosclerosis index could not be accurately calculated because of immature glomeruli in the rat pups on postnatal day 7 (P7); thus, we present representative images that show glomerulus atrophy with a dilated Bowman space and a shrunken tuft in hypoxic rat pups and no glomerulus atrophy in the normoxic rat pups. Tubular dilation was observed in the hypoxic group after HE staining (Figure 2A). PAS staining was used to evaluate tubular injury according to the loss of the brush border, tubular atrophy, tubular cast formation, tubular dilation, thickening of the tubular basement membrane, and sloughing of tubular epithelial cells. The hypoxic group exhibited significantly higher tubular injury scores than the normoxic group (Figure 2B). NGAL is a marker of kidney injury, and there was significantly higher expression of NGAL in hypoxic rat pups than in normoxic rat pups under immunohistochemistry and Western blot analysis (Figure 2C,D).

### 3.4. Effects of Neonatal Hypoxia on Body Weights and Protein Expression in the Kidney

**Figure 3:**
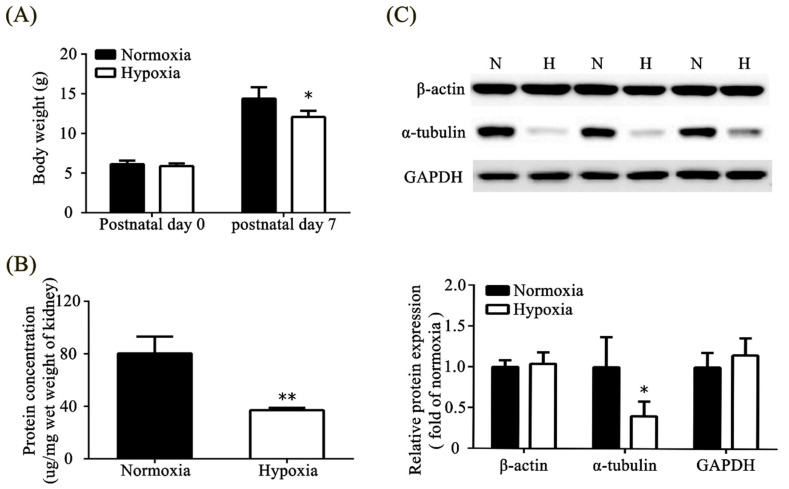
Hypoxia caused growth restriction and protein loss in the kidney. (**A**) Body weight on postnatal days 0 and 7. (**B**) Total protein concentration of the kidney tissue. (**C**) Western blot and quantitative analysis of different housekeeping proteins in the kidney tissue. The values are presented as mean ± standard deviation, * *p* < 0.05, ** *p* < 0.01, versus the normoxic group. The *p*-values were estimated via Mann–Whitney U test (*n* = 6–8). N, normoxia; H, hypoxia.

Rats in the hypoxic group weighed less than those in the normoxic group on P7 (Figure 3A). We extracted kidney tissue and found that hypoxia exposure significantly decreased the total protein concentration in the kidney tissue (Figure 3B). Among the three housekeeping proteins, β-actin, α-tubulin, and GAPDH, we found that the expression of α-tubulin was significantly decreased in the hypoxic group compared with that in the normoxic group (Figure 3C).

### 3.5. Effect of Neonatal Hypoxia on Fibrotic Change in the Kidney Tissue

**Figure 4:**
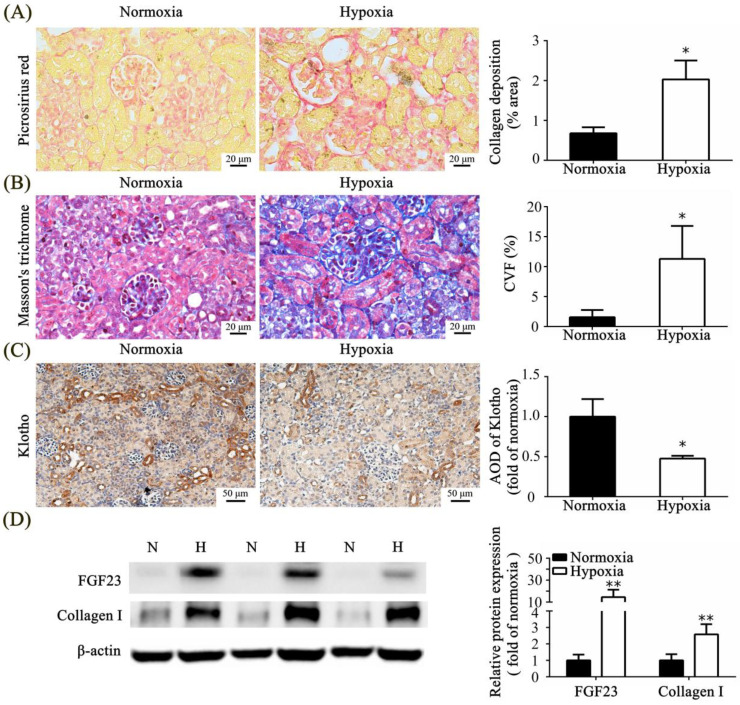
Neonatal hypoxia-induced kidney fibrosis. (**A**) Picrosirius red-stained images of the kidney tissue show collagen (red) under 400× magnification (scale bar, 20 μm) and quantification. (**B**) Masson trichome-stained images of the kidney tissue show fibrosis (blue) under 400× magnification (scale bar, 20 μm) and the CVF. (**C**) Representative images of klotho immunohistochemical staining in the kidney tissue under 200× magnification (scale bar, 50 μm) and quantitative analysis. (**D**) Representative images of FGF23 and collagen I immunoblotting in the kidney and quantitative analysis. The values are presented as mean ± standard deviation. * *p* < 0.05, ** *p* < 0.01, versus the normoxic group. The *p*-values were estimated via Mann–Whitney U test (*n* = 4–8). N, normoxia; H, hypoxia; CVF, collagen volume fraction; FGF23, fibroblast growth factor 23.

We used picrosirius red staining to calculate collagen deposition and found that the expression of collagen fibers was significantly increased in the hypoxic group compared with that in the normoxic group (Figure 4A).

The Masson trichrome-stained samples showed increased fibrosis in the hypoxic group compared with that in the normoxic group. There was a significant increase in CVF values in the hypoxic group compared with that in the normoxic group (Figure 4B). The results of these two staining methods were similar and verified, indicating the presence of kidney fibrosis in the hypoxic group. Rats in the hypoxic group exhibited significantly lower klotho expression than those in the normoxic group (Figure 4C). Western blot analysis revealed a significant increase in the expression of FGF23 and collagen I in the hypoxic group compared with that in the normoxic group (Figure 4D).

### 3.6. Effects of Neonatal Hypoxia on Oxidative Stress, Apoptosis, and Inflammation in the Kidney

**Figure 5:**
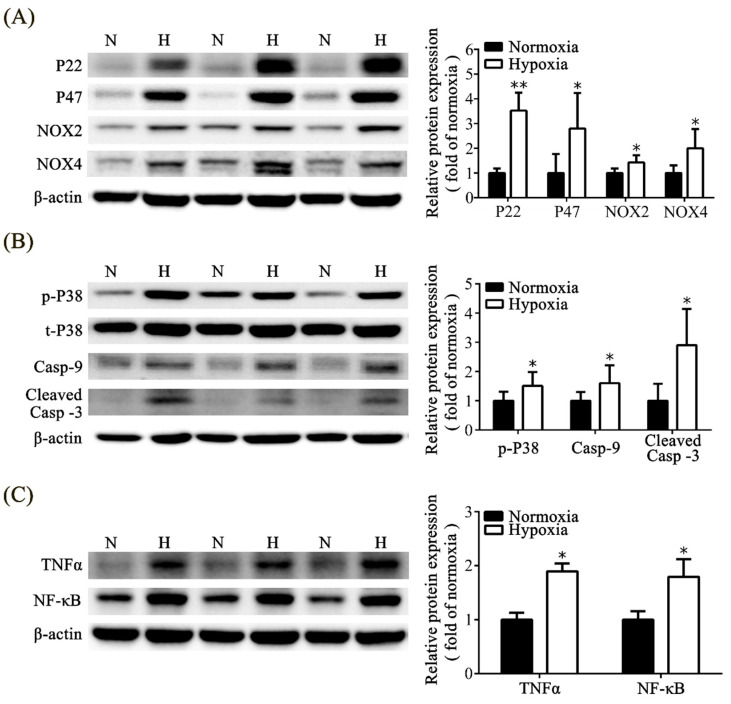
Hypoxic kidney injury involves oxidative stress, apoptosis, and inflammatory signaling. (**A**) Western blot analysis shows the oxidative-stress-related proteins, including P22, P47, NOX2, and NOX4. (**B**) Western blot analysis shows apoptosis-related proteins, including p-P38, Casp-9, and cleaved casp-3. (**C**) Western blot analysis shows the inflammatory-related proteins, including TNF-α and NF-κB. Quantitative analysis was performed for each blot. The values are presented as mean ± standard deviation. * *p* < 0.05, ** *p* < 0.01, versus the normoxic group. The *p*-values were estimated via Mann–Whitney U test (*n* = 6). N, normoxia; H, hypoxia. P22, nicotinamide adenine dinucleotide 3-phosphate (NADPH) oxidase subunit p22-phox; P47, NADPH oxidase subunit p47-phox; NOX2, NADPH oxidase 2; NOX4, NADPH oxidase 4; p-P38, phospho-p38 mitogen-activated protein kinase (Thr180/Tyr182); P38, p38 MAP kinase; Casp-9, caspase-9; cleaved Casp 3, cleaved-caspase-3; TNF-α, tumor necrosis factor-α; NF-κB, nuclear factor kappa-light-chain-enhancer of activated B cells.

We analyzed reactive oxygen species (ROS) in the kidney via Western blot analysis and found that the rats in the hypoxic group exhibited a significant increase in protein expressions, including P22, P47, NOX2, and NOX4, compared with those in the normoxic group (Figure 5A). To evaluate kidney apoptosis in the hypoxic animal model, we analyzed p-P38, caspase-9, and cleaved-caspase-3, which are involved in the mitogen-activated protein kinase pathway. Rats in the hypoxic group exhibited significantly increased expression of p-P38, caspase-9, and cleaved-caspase-3 compared with those in the normoxic group (Figure 5B). In addition, we discovered that inflammatory-related markers such as TNF-α and NF-κB were significantly higher in the hypoxic group compared to the normoxic group (Figure 5C).

## 4. Discussion

Our hypoxic animal model demonstrated that neonatal hypoxia during the first postnatal week induced oxidative stress, inflammation, apoptosis, and fibrosis of the kidney tissue, resulting in kidney injury.

Renal hypoxia is an important factor in kidney injury and progressive kidney disease, but most studies have been performed on adult animals. The most common cause of renal hypoxia is ischemia injury [22], and the effect of hypoxia on the kidneys of rats demonstrated reversible cell damage in immature kidneys [23]. Compared with local tissue hypoxia, systemic hypoxia did not alter total renal blood flow or perfusion and may trigger physiological processes for adaptation. The effect of hypoxia might be similar; however, systemic hypoxia, especially neonatal hypoxia, has been relatively less investigated. A neonatal AKI model showed that neonatal hypoxia for 24 h induced histopathological changes in the renal tissue, but the morphological changes in the kidney became insignificant after 72 h of hypoxia. Moreover, the mechanism of kidney injury was not mentioned [15]. Thus, we further investigated the effects of longer hypoxic exposure and hypoxic-induced kidney injury.

In our experiment, we analyzed the protein concentrations of HIF-1α and HO-1 in kidney tissue, both of which showed significantly increased expression in the hypoxic group compared with those in the normoxic group. The results of the blood gas analysis showed a significantly decreased pO_2_ level in hypoxic rats compared with that in normoxic rats, although the pO_2_ level in the normoxic group was lower than that in a previous study [7] (69.9 ± 6.5 mmHg) due to the samples possibly mixing with venous blood. Significant polycythemia was also observed in the hypoxic group. According to previous studies [24,25], persistent exposure to a low pO_2_ environment triggers the host to acclimate to physiological, metabolic, and cellular changes. Erythropoiesis is an early response to hypoxic exposure that can increase blood hemoglobin levels. HIF-1α accumulated under hypoxia and stimulated the expression of target genes, such as erythropoietin (EPO) and HO-1 [10]. HO-1 is thus upregulated and involved in iron and heme metabolism, reducing ferroptosis [26]. The development of polycythemia is associated with the coordination between the upregulation of EPO and induction of HO-1 during chronic hypoxia. These findings supported the validity of chronic hypoxia in our animal model. However, the significant elevation of the lactate level in the hypoxia group indicated tissue hypoxia, and the significant elevation of the creatinine level implied kidney injury. Although the creatinine level in the hypoxic rats did not meet the Kidney Disease Improving Global Outcomes clinical practice guidelines for acute kidney injury [27], we considered that it still influenced kidney tissue. Herein, we found obvious glomerulus atrophy with a dilated Bowman space and a shrunken tuft in the hypoxic group. Hypoxic rats also exhibited a higher grade of tubular injury than normoxic rats. Except for the morphological changes in kidney tissues, the NGAL level, regarded as a marker of kidney damage [28], was also significantly increased in the hypoxic group compared with that in the normoxic group. NGAL is a more sensitive marker than serum creatinine and can detect renal dysfunction at a very early phase [21]. These findings indicate that kidney injury was established under postnatal hypoxic conditions.

Our study showed that neonatal hypoxia significantly decreased body weight on P7, which is consistent with a finding from a previous study, which showed that hypoxia induced body growth retardation [1,4,6]. We did not explore the maternal effect on postnatal growth in hypoxia, but Mortola et al. [4] demonstrated that lactation and the behavior of hypoxic mothers did not affect the growth of rat pups. Another study compared the nutritional influences of rotated hypoxic dams and constant hypoxic dams and found no significant differences in body weight gain [1]. We did not analyze the kidney weight, but a previous study found that the ratios of kidney/body weights were similar in chronic constant hypoxia and control groups [1]. It is possible that due to the renal mass, the renal size and renal function were associated with the body’s metabolic demand [1]. An interesting finding is that the total protein concentration of kidney tissue in the hypoxic group was almost reduced by half compared with that in the normoxic group. Furthermore, α-tubulin expression was significantly lower in hypoxic rat pups compared to normoxic rat pups. These findings have not been reported in any other studies. However, we cannot explain the association and possible mechanisms based on the current evidence.

Renal fibrosis is a progressive wound-healing process of the kidney tissue that occurs in response to acute or sustained injuries [29]. Fibrosis is a pathological state in which excess fibroblasts deposited in extracellular matrices, including collagen and glycosaminoglycan, tend to compromise normal tissue functions [30]. We found that collagen fibers and CVF were significantly increased in the hypoxic group compared with those in the normoxic group based on picrosirius red staining and Masson trichrome staining of the kidney tissues. Klotho is a co-receptor for fibroblast growth factor-23 (FGF23) that modulates FGF23 signal transduction and is highly expressed in the kidney [31]. Downregulation or loss of renal klotho induces oxidative stress and renal fibrosis [31,32,33]. FGF23 is a bone-derived hormone involved in mineral metabolism through the kidney and parathyroid glands. Recent studies have demonstrated that upregulation of renal FGF23 in injured kidneys may increase myofibroblast activation and fibrogenesis [34,35]. Hao et al. [35] showed that FGF23 promotes glomerular and renal tubular fibrosis and the expression of renal fibroblasts. Based on previous evidence, we analyzed the expression of klotho and FGF23 in each group, and the rat pups in the hypoxic group exhibited significantly lower klotho expression with immunostaining than those in the normoxic group. In the injury-primed renal fibroblasts, under the absence of α-klotho, FGF23 induces phosphoinositide phospholipase C γ/calcineurin/nuclear factor of the activated T-cells signaling pathway by binding to fibroblast growth factor receptor 4, promotes the enhancement of the pro-fibrotic transforming growth factor β signaling pathway, and causes fibrogenesis [36]. Our results have similar findings. In our present study, we found decreased expression of klotho and increased expression of FGF23 in the kidney tissue of the hypoxia group. However, we cannot determine the pathologic pathway of signal transduction between FGF23 and klotho. We will explore the levels of phospholipase Cγ, nuclear factor of activated T-cells, and early growth response 1 in the hypoxic and normoxic rats in future work. In addition, the concentrations of FGF23 and collagen I were significantly increased in the hypoxic group compared with those in the normoxic group. These results suggest that neonatal hypoxia induces kidney fibrosis.

To investigate the mechanisms of kidney injury under hypoxic conditions, we analyzed several important proteins involved in different pathological pathways. Oxidative stress is an imbalance between cellular ROS levels and antioxidant enzymes that leads to pathological conditions [37]. Hypoxia induces oxidative stress and increases the production of ROS in the brain [8,38,39]. NADPH oxidases are the major sources of ROS in the cell [40,41]. The family of NADPH oxidases consists of seven isoforms (Nox1–5, Duox1, Duox2), and Nox4 is the predominant form in the kidney [40,42,43]. Nox1 and Nox2 are also expressed in the kidney. The NADPH oxidase is composed of the Nox protein and catalytic subunits, P22, P47, p67phox, and p40phox, and small GTP-binding proteins (G proteins Rac1 or Rac2) [44]. Upregulation of Nox4 has been linked to AKI [45] and chronic kidney disease [46], as well as transforming growth-factor-β-induced profibrotic responses [41]. Our findings of significantly increased expressions of Nox2, Nox4, P22, and P47 in the hypoxic group indicate an increase in oxidative stress in the kidney after neonatal hypoxia. The common pathway of the apoptosis cascade reaction is the activation of MAPK pathways, including p-P38, caspase 9, and cleaved-caspase 3 [47,48,49]. ROS also decreases mitochondrial membrane permeability and regulates Bax/Bcl2 in mitochondria to activate caspase-9 and cleaved-caspase-3, resulting in apoptosis [37]. In our experiment, the hypoxic rats exhibited significant upregulation of p-P38, caspase-9, and cleaved-caspase-3, which confirmed activation of apoptosis in the kidney tissue. The expression of inflammatory markers, including TNF-α and NF-κB, in the kidney tissue was upregulated in hypoxic rat pups. Hypoxic stimulation induced the expression of TNF-α, which may be associated with early renal injury [50]. TNF-α also induces Nox4 [51]. Based on previous studies [26,52,53], activation of the NF-κB pathway is important for the initiation and progression of inflammation and is induced by hypoxia. In some animal models of kidney injury, NF-κB activation has been reported in renal disease, inflammation, and renal fibrosis [53,54,55,56].

The limitations of this study are as follows. Urine sampling from rat pups was difficult; hence, the relevant urine biomarkers for kidney injury could not be analyzed in our experiment.

In conclusion, we demonstrated that neonatal hypoxia causes growth restriction and morphological changes such as glomerular atrophy and tubular injury in the kidney tissue. We also confirmed kidney damage through the pathways of oxidative stress, inflammation, apoptosis, and fibrosis after hypoxia during the first week of life. Only one week of hypoxia was used to explore the molecular mechanisms of early kidney injury due to hypoxia. Thus, further investigation is needed to explore whether prolonged, constant hypoxia leads to progression from AKI to chronic kidney disease.

## Figures and Tables

**Table 1:** Parameters of blood gas analysis.

Parameter/Group	Normoxia	Hypoxia
pH	7.38 ± 0.02	7.35 ± 0.06
Glucose (mg/dL)	118.20 ± 21.81	124.67 ± 15.04
Creatinine (mg/dL)	0.51 ± 0.03	0.71 ± 0.09 *
Lactate (mmol/L)	2.14 ± 0.69	4.12 ± 0.45 *
pCO_2_ (mmHg)	49.18 ± 3.39	50.83 ± 10.19
pO_2_ (mmHg)	56.68 ± 4.09	27.77 ± 6.72 *
cTCO_2_ (mmol/L)	30.82 ± 1.25	29.00 ± 1.74
cSO_2_ (%)	87.94 ± 2.53	46.93 ± 17.41 *
Sodium (mmol/L)	128.00 ± 0.71	127.00 ± 1.73
Potassium (mmol/L)	6.84 ± 0.51	11.17 ± 1.44 *
Chloride (mmol/L)	97.60 ± 2.19	102.00 ± 1.73 *
Calcium (mmol/L)	1.49 ± 0.08	1.36 ± 0.07
Hematocrit (%)	22.00 ± 1.22	35.67 ± 0.58 *
cHgb (g/dL)	7.50 ± 0.41	12.17 ± 0.21 *
cHCO_3_^−^ (mmol/L)	29.32 ± 1.16	27.43 ± 1.46
BE(ecf) (mmol/L)	4.26 ± 1.09	1.77 ± 0.55 *
BE(b) (mmol/L)	3.76 ± 0.96	1.03 ± 0.32 *
AGapK (mmol/L)	7.80 ± 1.64	8.60 ± 1.40
AGap (mmol/L)	1.00 ± 2.35	−2.33 ± 0.58

The values are presented as the means ± SD, *p*-values were estimated via Mann-Whitney U test, * *p* < 0.05, versus the NC group (*n* = 3–5). pCO_2_: partial pressure of carbon dioxide; pO_2_: partial pressure of oxygen; cTCO_2_: concentration of total carbon dioxide; cSO_2_: concentration of oxygen saturation; cHgb: concentration of hemoglobin; cHCO_3_^−^: concentration of bicarbonate; BE(ecf): base excess of the extracellular fluid; BE(b): blood base excess; AGapK: anion gap potassium; and AGap: anion gap.

## Data Availability

Not applicable.
